# Modelling urea cycle disorders using iPSCs

**DOI:** 10.1038/s41536-022-00252-5

**Published:** 2022-09-26

**Authors:** Claire Duff, Julien Baruteau

**Affiliations:** 1 Genetics and Genomic Medicine Department, Great Ormond Street Institute of Child Health, University College London, London, UK; 2 National Institute of Health Research Great Ormond Street Biomedical Research Centre, London, UK; 3 Metabolic Medicine Department, Great Ormond Street Hospital for Children NHS Foundation Trust, London, UK

**Keywords:** Induced pluripotent stem cells, Experimental models of disease, Metabolic disorders, Mechanisms of disease, Stem-cell differentiation

## Abstract

The urea cycle is a liver-based pathway enabling disposal of nitrogen waste. Urea cycle disorders (UCDs) are inherited metabolic diseases caused by deficiency of enzymes or transporters involved in the urea cycle and have a prevalence of 1:35,000 live births. Patients present recurrent acute hyperammonaemia, which causes high rate of death and neurological sequelae. Long-term therapy relies on a protein-restricted diet and ammonia scavenger drugs. Currently, liver transplantation is the only cure. Hence, high unmet needs require the identification of effective methods to model these diseases to generate innovative therapeutics. Advances in both induced pluripotent stem cells (iPSCs) and genome editing technologies have provided an invaluable opportunity to model patient-specific phenotypes in vitro by creating patients’ avatar models, to investigate the pathophysiology, uncover novel therapeutic targets and provide a platform for drug discovery. This review summarises the progress made thus far in generating 2- and 3-dimensional iPSCs models for UCDs, the challenges encountered and how iPSCs offer future avenues for innovation in developing the next-generation of therapies for UCDs.

## Introduction

**Fig. 1:**
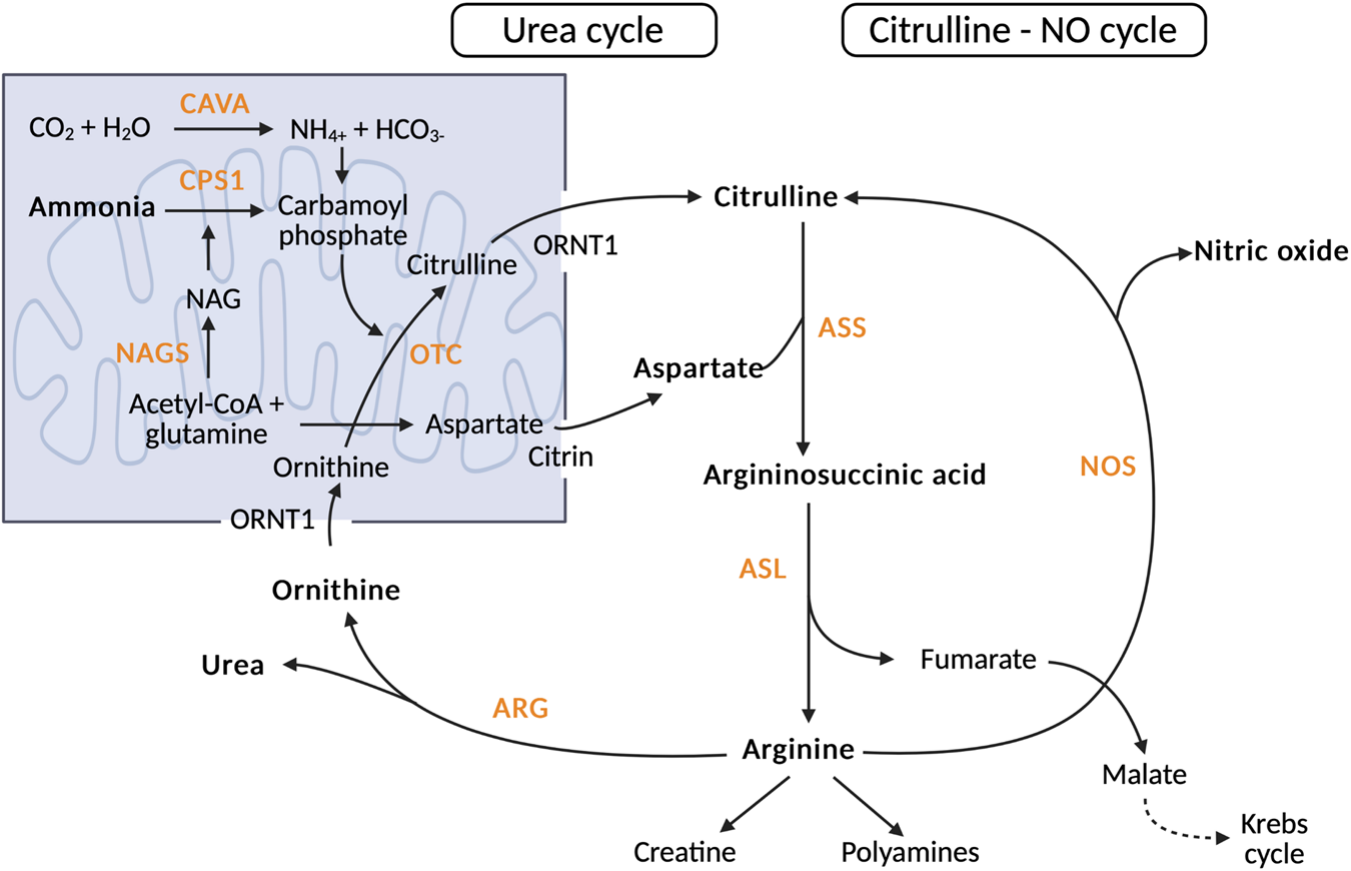
The urea and citrulline-nitric oxide cycles. Ammonia is converted to urea via five consecutive enzymatic reactions within the liver. The urea cycle begins in the mitochondria (blue box), where ammonia and bicarbonate are converted into carbamoyl phosphate. Carbamoyl phosphate requires allosteric activation by N-acetylglucosamine (NAG). Ornithine transcarbamylase (OTC) then catalyses formation of citrulline from carbamoyl phosphate and ornithine. Citrulline is channelled out of the mitochondria via the ornithine transporter (ORNT1), whilst citrin transports aspartate from the mitochondria to the cytoplasm. Argininosuccinate synthetase then enables the synthesis of argininosuccinate from citrulline and aspartate. Argininosuccinate is subsequently broken down into arginine and fumarate by argininosuccinate lyase, with fumarate directed to the Krebs cycle. Arginine is broken down into urea and ornithine by arginase. Ornithine is transported into the mitochondria by the ORNT1 transporter. CA5A carbonic anhydrase VA, CPS1 carbamoyl phosphate synthetase 1, NAG N-acetyl glutamate, NAGS N-acetyl glutamate synthase, OTC ornithine transcarbamylase, OTC1 ornithine transporter, ASS argininosuccinate synthetase, ASL argininosuccinate lyase, ARG arginase, NOS nitric oxide synthase.

Urea cycle disorders (UCDs) encompass a set of rare inherited disorders characterised by defects in one of the urea cycle enzymes or transporters that contribute to detoxify nitrogen waste i.e., ammonia. Ammonia is produced by the deamination of amino acids and is a highly neurotoxic molecule. The urea cycle pathway is fully expressed in the liver (Fig. 1), precisely in periportal hepatocytes following a specific metabolic zonation^1^. Six enzymes and two transporters have been identified as causing primary UCDs (Table 1), with an estimated incidence of 1:35,000 births in the USA and Western Europe^2^. UCDs cause hyperammonaemic decompensation with cerebral oedema, resulting in a range of specific symptoms from loss of appetite, vomiting, dizziness, lethargy and seizures, to coma leading to death^3^. Unrecognised chronic hyperammonaemia causes severe developmental delay and behavioural difficulties^4–6^. Gold standard management relies on protein-restricted diet, ammonia scavenger drugs, arginine supplementation and close monitoring of plasma ammonia, amino acids and nutritional status. Liver transplantation is currently the only curative option^7,8^. Gene therapy was developed targeting ornithine transcarbamylase deficiency in the late 1990s but the immunogenicity of the viral adenoviral vector led to tragic consequences^9^. However, in more recent years, safer approaches have been translated using adeno-associated virus (AAV) derived vectors (NTC02991144)^10^ or mRNA encapsulated lipid nanoparticles (NTC04442347)^11^. An early phase clinical trial of AAV gene therapy for ornithine transcarbamylase deficiency in adults has recently shown promise as an alternative disease-modifying therapy^12^. These diseases are rare, which precludes an optimal understanding of the complex pathophysiology of the disease, especially in the brain^13–17^. Indeed, the spectrum of clinical severity of the same UCD can be highly variable between individuals^13,18,19^. Yet, despite a common cerebral toxicity as a result of hyperammonaemia, there are pathophysiological specificities, which remain only partially understood, such as arginase deficiency and dysmyelination^20^, argininosuccinic aciduria and oxidative stress^21–24^, or Lysinuric Protein Intolerance and impaired phagocytosis^25^. Some mouse models fail to recapitulate the human phenotype, such as the hypomorphic *Spf*^*ash*^ mouse modelling ornithine transcarbamylase deficiency (OTCD), where a high residual ureagenesis capacity prevents hyperammonaemia, UCDs’ cardinal feature, under standard diet. In the *Spf*^*ash*^ mouse, hyperammonaemia is observed with a protein-enriched diet meaning that only partial OTCD is recapitulated^26,27^. In addition, traditional cell lines usually derived from immortalised cancer cell lines, have their own limitations with metabolic alterations, leading to a need for alternative modelling methods^28^. The parallel emergence of induced pluripotent stem cells (iPSCs) and CRISPR/Cas9 editing have provided the opportunity to model these disorders in vitro giving the potential to uncover novel mechanisms of action and new therapeutics, whilst overcoming some of the limitations posed by other models. iPSCs are derived from differentiated adult somatic cells and are characterised by their ability to propagate indefinitely and their capacity to differentiate into any cell type of the human body^29–31^. This review aims to discuss the ongoing progress and remaining limitations that have occurred in modelling these rare disorders using iPSCs.

**Table 1:** Summary of the Urea Cycle Disorders.

Defect	Gene	Inheritance	Mutated protein	OMIM	Prevalence^2^	Main presentation
CAVA deficiency	*CA5A*	Recessive	Carbonic anhydrase VA	615751	<1/2,000,000	Acute encephalopathy^114^
NAGS deficiency	*NAGS*	Recessive	N-acetylglutamate synthase	237310	<1/2,000,000	Hyperammonaemia^115^
CPS1 deficiency	*CPS1*	Recessive	Carbamoyl phosphate synthase	237300	1/1,300,000	Hyperammonaemia^116^
OTC deficiency	*OTC*	X-linked	Ornithine transcarbamylase	311250	1/56,500	Hyperammonaemia^117^
Citrullinemia 1	*ASS*	Recessive	Argininosuccinate synthase	215700	1/250,000	Hyperammonaemia^60^
Argininosuccinic aciduria	*ASL*	Recessive	Argininosuccinate lyase	207900	1/110,000	Hyperammonaemia, arterial hypertension, developmental delay, chronic liver disease^21^
Arginase	*ARG1*	Recessive	Arginase type 1	207800	1/950,000	Hyperammonaemia, diplegic spasticity^118^
Hyperornithinaemia, hyperammonaemia, homocitrullinuria (HHH) syndrome	*SLC25A15/ORNT1*	Recessive	Ornithine-citrulline antiporter	238970	<1/2,000,000	Hyperammonaemia, diplegic spasticity^119^
Citrin deficiency	*SLC25A13*	Recessive	Mitochondrial aspartate-glutamate carrier	605814, 603471	1/150,000	Hyperammonaemia, neonatal cholestasis, failure to thrive, dyslipidaemia, chronic liver disease^120^
Lysinuric protein intolerance (LPI)	*SLC7A7*	Recessive	Dibasic cationic amino acid (CAA) transporter	222700	<1/2,000,000	Hyperammonaemia, failure to thrive, short stature, renal disease^14,25^

## UCD-derived iPSCs: purpose and advantages

One of the challenges in modelling liver monogenic diseases in vitro is often the lack of reliable models. Whilst immortalised human liver cancer cell lines are commonly used, such as human hepatocyte-derived Huh7 or HepG2 cell lines as surrogates of human primary hepatocytes, they differ from them in many aspects. Metabolic enzymes are invariably expressed at suboptimal levels when compared to primary hepatocytes^32–34^. In addition, immortalised cancer cell lines are often immature, have dysfunctional apoptotic pathways and have limited genotypic variability^35^. Primary hepatocytes are considered the gold standard and provide a more biologically relevant model, recapitulating several features of a healthy liver cell in vivo^36,37^. However, these cells are challenging to isolate, have limited proliferative capacity, limited viability in vitro and rapidly lose hepatic function, preventing them being maintained in culture long-term^38–41^. Foetal and adult human stem cells can as well be used although they can be technically challenging to isolate and culture^42,43^. Therefore, in the last few years, iPSCs have provided an alternative strategy to model inherited metabolic diseases, particularly UCDs (Table 2). These cells have the capacity to differentiate into all three germ layers, enabling the prospect of generating any cell type in vitro^29^. In addition to averting the ethical issues associated with embryonic stem cells, one major benefit of iPSCs is the opportunity to derive cells from individuals encompassing a spectrum of genetic backgrounds, generating patients’ avatars. Protocols are constantly being optimised with the aim to produce fully mature and functional cells. These protocols utilise growth factors and small molecules which replicate in vivo development. Differentiated iPSCs can be used for an organ-on-a-chip approach^44–46^. Previous work in other fields, such as cancer, has demonstrated that organ-on-a-chip approaches can yield organ-level function superior to other 2D/3D in vitro cell models^47,48^. In addition, unlike 2D culture systems, organ-on-a-chip can elicit more physiologically relevant pharmacological responses, allowing for more accurate predictions of drug efficacy, toxicity and pharmacokinetics^49^. Further, recent studies have produced multi-organ platforms, where mutiple organs are integrated on a single device, which allows modelling of the more complex in vivo environment^50^.

**Table 2:** Advantages and Pitfalls of Experimental Approaches in Disease Modelling.

Model	Pros	Cons
Primary cells^121–123^	• Conserved functional properties • Incorporate all cell types in heterogeneous tissue	• Limited availability / limited proliferation in vitro (cell type-dependent) • Different genotype depending on donor – lack of reproducibility • Large variation in quality/viability • Difficult to maintain cell functionality in vitro • Limited genetic manipulation
Human cell lines^122–124^	• Expandable • Similar transcriptional markers and functional properties to human primary cells	• Immortalised/proliferative • Aneuploid • Low clonal efficiency • Genetic manipulation can be limited • Batch-to-batch variations • Reduced functional resolution • No disease-enabling genetic background
Human adult stem cells and human foetal liver^42,43,125^	• Share similar properties to human primary cells • Proliferative	• Difficult to extract • Difficult to culture
Human induced pluripotent stem cells^79,126,127^	• Can differentiate into any cell type • Easy to genetically manipulate	• Variable differentiation efficiency • Functionally immature • Polyhormonal cells
Organ-on-a-chip^44–46,49^	• More physiologically relevant pharmacological responses • Models a more complex in vivo environment	• Expensive • Cultures are challenging
Genetically modified models^128,129^	• In vivo • Some anatomical and physiological similarities • Allow proof of concept studies	• Some anatomical and physiological differences • Can be expensive and labour intensive with larger animal models
Xenograft models^15,130,131^	• In vivo • Interventions can be implemented at optimal time • Multiple therapies can be tested on the same biopsy	• Xenograft may not be representative of the original human cells in their native state • Results can be influenced by host’s physiology • Suboptimal surrogate of human immune system

Utilising iPSCs can also have advantages over in vivo models, particularly when considering the ethical principles of the three Rs (replacement, reduction and refinement), which oversee animal research (https://nc3rs.org.uk/the-3rs)^51^. In vivo animal models can be labour intensive and expensive, and the resultant animal may fail to recapitulate several features of the human phenotype. In addition, the translation of drug trials to humans from animal models can be unsuccessful due to species-specific biological responses, contributing to the translational “valley of death” and high failure rates in the approval of novel therapies^52^. With iPSCs, mutliple cell lines derived from the same patient can be cultured simultaneously in a much smaller space, with each line having the capacity to be differentiated into several tissue types, which can also reduce the time and labour required. iPSCs are therefore more easily scalable and accessible than animal models. Experiments can be compared across different tissue types to cells that are all derived from the same genetic background. Furthermore, pharmacology and toxicology assessments can be made patient-specific to avoid possible adverse side effects.

## UCD-derived iPSCs: state of the art

In the last decade, iPSCs have been increasingly used as experimental models to (i) identify pathogenicity of variant of uncertain significance (VUS), (ii) study disease mechanisms, (iii) develop and screen therapeutic strategies in vitro, with a potential for in vivo applications and iPSC transplantation to treat animal models (Table 3). As the urea cycle is only able to express in its totality in the liver, UCDs-derived iPSCs are frequently differentiated into hepatocyte-like cells. These cells are functionally assessed and compared to isogenic control lines, often generated via CRIPSR/Cas9 editing^35,53^.

**Table 3:** Summary of studies performed using iPSCs to model UCDs.

Donor Information						Experimental Design		
UCD subtype	Cell source	Genotype	Age	Sex	Clinical severity	Purpose of study	Differentiation	Control line used
Ornithine transcarbamylase deficiency	PBMCs PBMCs Fibroblasts Fibroblasts	Deletion of 3 to 9 exons in OTC gene Hemizygote mutation (c.663 + 2 T > G); XXY c.386 G > A Exon 6, c.548 A > G [p.Tyr183Cys]; exon 3, c.274 C > T [p.Arg92*]	4 days 3 days 9 months Neonatal; 6 years	M M M M, F	Hyperammonaemia Multiple organ failure Hyperammonaemia – liver transplantation Fatal neonatal hyperammonaemia; fatal acute liver failure	Generation of stem cell line from patient Generation of stem cell line from patient Assessment of genetic and phenotypic markers in both patient and corrected differentiated cells In vitro *model of* liver disease	Embryoid bodies Embryoid bodies Hepatocyte-like cells Hepatocyte-like cells	N/A^55^ N/A^56^ CRISPR/Cas9 corrected iPSCs^53^ Healthy donors^57^
Argininosuccinate synthetase deficiency (Citrullinemia type 1)	Fibroblasts PBMCs	p∼G390R (c.1168 G > A) Compound heterozygous: Exon 6, c.364-2 A > G, p. G259*; Exon 13, c.910 C > T, p. R304W	N/S 5 years	N/S F	Neonatal hyperammonaemia Neonatal hyperammonaemia	Generating and characterising the hepatic organoid (eHEPO) culture system Assessment of differentiated patient iPSCs, found partial recapitulation of patient phenotype	Hepatic organoids Hepatocyte-like cells	Healthy donors^64^ Healthy donors^63^
Argininosuccinate lyase deficiency	Fibroblasts	Compound heterozygous at exon 7 and 11 Compound heterozygous at exon 7 and 11	N/S N/S	N/S N/S	N/S N/S	Assessment of effect of ASL loss on endothelial cells Assessment of loss of ASL on osteoblast differentiation	Endothelial cells Osteogenic	Healthy donors^67^ Helper-dependent adenovirus system corrected iPSCs^68^
Arginase deficiency	Fibroblasts	N/S	1 F – 5 years Other lines N/S	1 M, 2 F	Developmental delay, microcephaly, spasticity (1 F, 5 years), other lines N/S	Implement a CRISPR/Cas9-based strategy to genetically modify and restore arginase activity	Hepatocyte-like cells	Healthy donor^69^
Citrin or aspartate/glutamate carrier	Fibroblasts	Compound heterozygous (851del4; IVS16ins3kb)	1-year old	M	Jaundice and liver failure	Assessment of differentiated patient-derived iPSCs – show that aberrant mitochondrial b-oxidation may give rise to fatty liver in citrin deficient patients	Hepatocyte-like cells	Healthy donor^75^
Carbamoyl phosphate synthetase 1 (CPS1) deficiency	Fibroblasts	E832X; D914H (c.4162-2 A > G); V204F	Neonatal	M, M, F	Neonatal hyperammonaemia	Assessment of differentiated patient-derived iPSCs in comparison to CRISPR/Cas9 corrected controls.	Hepatocyte-like cells	Human embryonic stem cells^54^

### CPS1 deficiency

This disease has been investigated using patient-derived iPSCs, enabling the results to be compared to the clinical phenotype of the affected individuals^54^. In this study, patient iPSC lines were genetically modified via CRISPR/Cas9 to constitutively express human codon optimised *CPS1* from the AAVS1 safe harbour site and then differentiated into hepatocytes. The study aimed to assess the therapeutic potential of this approach. Despite successful differentiation, interestingly, the corrected cells failed to metabolise ammonia any more effectively than the patient cell lines. This unexpected outcome was proposed to be due to transgene promoter methylation and resultant transcriptional silencing in the undifferentiated cells. This study emphasises the requirement for thorough quality control and functional assessment of iPSCs prior to use for downstream experiments.

### Ornithine transcarbamylase deficiency

Some studies develop iPSC lines from peripheral blood mononuclear cells (PBMCS), such as with neonatal-onset OTCD patients^55,56^ (Table 3). These studies show proof of concept that iPSCs can be generated from newly diagnosed patients and thus provide a model to develop a personalised therapeutic approach. Zabulica et al. functionally characterised iPSC-derived hepatocytes compared to isogenic controls and showed an isolated reduced *OTC* expression from day 8 of differentiation onwards and a significant reduction in ureagenesis. Importantly, the study identified only three potential off-target modifications of CRISPR/Cas9 editing, located in intronic or intergenic regions and highly likely to be biologically inactive^53^. Laemmle et al. investigated the role of aquaporin 9 (AQP9), a membrane channel protein that facilitates transit of water, glycerol, and urea, in OTCD iPSC-derived hepatocytes. In healthy control, AQP9 overexpression enabled increased ureagenesis and a phenotype closer to the in vivo environment. OTCD iPSC-derived hepatocytes recapitulated the hepatic manifestation of the patient phenotype with reduced ureagenesis and OTC activity. This phenotype was corrected after lentiviral gene therapy^57^.

### Citrullinaemia type I

More than 130 private mutations of the *ASS1* gene have been described^58–60^. The identification of variant of uncertain significance (VUS) is a large-scale problem in the era of high-throughput genomics^61^. The use of patients’ iPSCs can provide a unique model to perform functional studies to assess pathogenicity of a mutation with the additional possibility of screening personalised therapeutics. iPSCs carrying the disease-specific mutations can be artificially created and then used for functional studies as highlighted in citrullinaemia type I (CTLN1)^62^. Yoshitoshi-Uebayashi et al. differentiated iPSCs from a citrullinaemic patient and isogenic controls into functional hepatocytes, but failed to fully recapitulate a UCD phenotype, potentially due to incomplete hepatocyte differentiation. Baseline ammonia and urea levels between patient and control lines were not different but an ammonia challenge assay exhibited significantly reduced ureagenesis in *ASS1*^*-/-*^ cells. Interestingly, the study highlighted significantly increased levels of several tricarboxylic acid (TCA) cycle-related metabolites such as pyruvate, succinate and aspartate, shedding light on aspartate accumulation caused by ASS deficiency and diversion towards TCA cycle^63^.

Akbari et al. differentiated iPSCs from a CTLN1 patient into endoderm-derived hepatic organoids, following a 2 week-differentiation protocol and prolonged expansion over a year^64^. The liver organoids for both wild-type and patient cells demonstrated similar architecture and appropriate hepatocyte differentiation confirmed by expression of hepatocyte markers, e.g., HNF4A and CK19 and functional assays, e.g., albumin production, low density lipoprotein (LDL) uptake and glycogen storage. Transcriptomic analysis was similar between wild-type and patient 3D cultures indicating that *ASS1* mutation did not interfere with differentiation ability. Functionally, the CTLN1 organoids had significantly increased ammonia, with overexpression of wild-type *ASS1* restoring arginine levels to wild-type levels and partially rescuing ureagenesis. These data demonstrated that hepatic organoids can recapitulate several features of the disease phenotype that can be alleviated with therapies targeting gene function^64^.

### Argininosuccinate lyase deficiency

Argininosuccinate lyase deficiency (ASLD) is an atypical UCD due to a systemic phenotype affecting multiple organs^4,21^. Hence ASLD is a challenging disease to treat as liver-targeted gene therapy corrects ureagenesis but does not alleviate other phenotypic symptoms, especially within the brain^65^ or endothelial cells^66^. ASLD iPSCs have been differentiated into other tissues than hepatocytes for investigating ASLD pathophysiology, to address the consequences of arginine and downstream metabolite nitric oxide (NO) deficiencies. Kho et al. demonstrated that ASLD can present as endothelial-dependent hypertension. The authors used multiple models, in addition to clinical studies, including iPSC-derived endothelial cells^67^. ASLD iPSCs did not differentiate as efficiently into endothelial cells as the controls, indicating that ASL participates in vasculogenesis via NO production. ASLD iPSC-derived endothelial cells had decreased levels of intracellular NO. The cells, when injected into immunodeficient (NOD/SCID/IL2Rγ − ) mice, demonstrated a decreased ability to establish blood capillaries and arterioles in vivo. The data, combined with their results from a human clinical study, mouse model and in vitro cell models, demonstrated that endothelial dysfunction is the main cause of hypertension in ASLD. This shows the ability of iPSCs to be used to model disease and validate findings from other in vitro and in vivo models^67^. Jin et al. investigated the effect of ASLD in human osteoblasts, using ASLD iPSCs compared to two isogenic controls^68^. The ASLD-iPSC derived osteoblasts had decreased mineralisation capacity, significantly reduced expression of glycolytic genes such as *SLC2A1*, *PKM* and *LDHA*, or GLUT1 protein suggesting that ASLD leads to the deficient glycolysis. Treatment with NO donor S-nitroso-N-acetylpenicillamine (SNAP) during osteoblast differentiation led to an upregulation of the glycolytic genes. These findings that ASLD-iPSCs have a decreased ability to differentiate into osteoblasts and impaired glycolysis was confirmed in two additional animal models and murine osteoblastic cell lines, highlighting the reliability of iPSCs in investigating pathophysiology and identifying new therapeutic targets^68^.

### Arginase-1 deficiency

Hui et al. studied CRISPR/Cas9-edited iPSCs lines from three patients with arginase-1 deficiency, in an attempt to restore arginase-1 activity. Corrected iPSCs were compared to unedited controls and exhibited restoration of arginase expression and ureagenesis from functional arginase. The three corrected lines were successfully differentiated over 21 days into hepatocytes, alongside their uncorrected counterparts and a control line. Off-target editing was evaluated by sequencing specific sites identified in silico being at risk of off-target effect. Neither insertion nor deletion had occurred^69^. Such studies provide a strategy for restoring gene function by editing paving the way for genetically modified cell therapies to treat UCDs. Arginase-1 deficiency has also been studied using iPSC-derived hepatocyte-like cells, as well as iPSC-derived macrophages. Utilising PiggyBac technology alongside CRISPR/Cas9, exons 7 and 8 of *ARG1* were reincorporated into iPSCs derived from an arginase-1 deficient mouse model^70^, a knockout model where mutant animals die at about 2 weeks of age, in order to assess whether *ARG1* function could be restored. Successful gene repair was confirmed, but ureagenesis remained suboptimal compared to adult hepatocytes, probably caused by insufficient hepatocyte differentiation. In comparison, iPSC-derived macrophages showed corrected arginase function that could be explained by a more ready differentiation into a functionally mature state^71^. The study demonstrated how in vitro research can be developed using iPSCs and CRISPR/Cas9 to conduct follow-up investigations in various differentiated cell types. Differentiated cells can then be transplanted in vivo to enable further maturation and long-term repopulation of hepatocytes, for instance^72,73^. The same group also used transcription activator-like effector nucleases (TALENs) to correct the same defect and again, differentiated these cells into hepatocyte-like cells. These cells were then transplanted back via intravenous infusion into 12-week-old arginase-1 deficient mice, in a tamoxifen-induced *ARG1* knock-out mouse model. iPSC-transplanted arginase-1 deficient mice survived an additional week compared to untreated knock-out littermates without reaching statistically significant increase of survival. The treated mice had increased arginase-1 expression but ureagenesis was not comparable to that of wild-type controls. The inability to fully rescue the phenotype may be caused by the inability for transplanted cells to repopulate the periportal hepatocytes where the urea cycle is expressed, suboptimal hepatocyte differentiation, low engraftment or immunological rejection of transplanted cells^74^.

### Citrin deficiency

Sin et al. differentiated iPSC from a patient with citrin deficiency into hepatocytes. Functional assessment confirmed that patient cells did not exhibit ureagenesis. Genes involved in mitochondrial beta-oxidation were downregulated in the patient cells, with abnormal mitochondrial structure, leading to significantly higher levels of cellular triglycerides and lipid granule levels compared to the wild-type cells. The work was further validated by a complementary *Slc25a13* knockout mouse model, which recapitulated some, although not all, of the patient phenotype^75^.

## Limitations of iPSCs for UCD research

iPSCs models present intrinsic limitations, particularly for therapeutic application and clinical translation.

One of the significant challenges faced by iPSCs research is, despite ongoing improvements in differentiation protocols, the immaturity of iPSC-derived cells, which remain closer to foetal rather than their fully differentiated counterparts^76^. This has been demonstrated in modelling UCDs, where both wild-type and mutated immature differentiated hepatocytes failed to achieve equivalent functionality with that of in vivo hepatocytes^53,63,74^. This means that results must be interpreted with caution for UCDs and particularly when modelling disease specificities associated with age. Furthermore, differentiation efficiency and reproducibility can vary due to differing protocols, genetic backgrounds, passage number and epigenetic memory^77–79^. iPSCs can also acquire mutations that confer a growth advantage, particularly p53 mutations, advocating for thorough genetic characterisation prior to translation to the clinic^80^. With CRISPR/Cas9 mediated reprogramming, off-target effects that could adversely alter the cell function need to be carefully assessed^81^. However, efforts have been made to improve identification of off-targets using in vitro assays, including circularisation for in vitro reporting of cleavage effects by sequencing (CIRCLE-seq)^82^ and selective enrichment and identification of tagged genomic DNA ends by sequencing (SITE-seq)^83^, as well as improving the computational platforms used to design sgRNAs^84^. Another approach that can reduce the number of off-target effects is base editing, whereby a catalytically inactive Cas9 is employed together with cytidine deaminases to introduce site specific DNA mutations/corrections without the requirement for a double-strand break^85^.

Another iPSC limitation of use in research is that the cell population produced may not be representative of more complex in vivo architecture and morphology. Differentiation protocols often make a single cell type, whereas in vivo hepatocytes, for instance, are in close proximity to non-parenchymal cells such as stellate cells, Kupffer cells and endothelial cells, facilitating crosstalk between parenchymal and non-parenchymal cells. This heterogeneous population, in addition to nutrients, growth factors and vasculature all work together to support the genetic expression and viability of the hepatocytes^86^. However, there are steps to overcome this limitation with the use of co-cultures – for instance, co-culturing hepatocytes with endothelial and stromal cells aids maturation of hepatocytes^87^. In the context of UCDs, urea cycle activity and ammonia metabolism are confined to zone 1 located hepatocytes nearest the portal vein, as part of the regulated compartmentalisation of the hepatic lobule^88^. However, at present, only partial zonation is able to be achieved, which may influence how well ammonia metabolism can be modelled^89^. Nevertheless, these co-cultures cannot completely recapture the complexity of the in vivo environment.

A safety issue for clinical translation is the risk of teratoma formation^90^. This is particularly a consideration for UCDs as these diseases have occasionally been associated with hepatocellular carcinoma^4,91,92^. There is a risk of potential tumorigenicity and malignant tumours if transplanted cells are contaminated with undifferentiated iPSCs. However, there are treatments that could potentially treat these teratomas if they occur^93^. There are also purification methods such as flow cytometry sorting and small chemical molecules that initiate death of undifferentiated iPSCs which could help lower the risk of tumorigenicity. However, the effectiveness of such methods has yet to be fully elucidated^94^. Autologous iPSC therapies effectively eliminate the potential for immune rejection. The generation of clinical grade autologous iPSC-derived cells entails high production costs associated with a rigorous quality control system. As the generation of the patient-specific tissue of interest can take several months, this timeframe might not be suitable for neonatal-onset UCD patients, which require an urgent liver replacement strategy^95^. Therefore, an alternative approach is to use allogeneic iPSC-derived cell sources which would enable biobanking a limited number of approved iPSCs from assorted human leukocyte antigen (HLA)-homozygous donors that would match the bulk of the population^96^. These cells would have all undergone regulatory clearance and have been thoroughly tested meaning that the cells could be more readily used to treat patients who require timely intervention. However, patients would be required to undergo lifelong immunosuppression treatment.

For clinical trial applications, ethical consent should be obtained freely. For children, eligibility should be considered only if there is minimal or low risk with potential for direct benefit, and the procedure is as favourable as available alternatives^97^. Regulatory guidelines such as the US Food and Drug Administration (FDA)^98^, the European Medicines Agency (EMA) (https://www.ema.europa.eu/en/documents/scientific-guideline/guideline-human-cell-based-medicinal-products_en.pdf) and the International Society for Stem Cell Research are in place to ensure that patient safety is at the forefront^99–101^. Adhering to these guidelines, alongside potential intellectual property and patent considerations, can add substantial time and effort to the setting up and delivery of iPSCs studies in accordance with Good Clinical Practice. Additionally, scalable technology to meet regulatory standards remains a challenge for generating genetically modified iPSCs for cell therapy, as it requires special equipment alongside skilled operators^102–104^. Thus, these limitations may prevent potential therapeutic treatments from reaching patients due to prohibitive cost and time requirements.

## Future directions

iPSC differentiation provides a unique opportunity to study a genetic disease in a specific genetic and epigenetic background in vitro and screen for therapeutic candidates. iPSCs have improved our knowledge of the pathophysiological mechanisms of several diseases, alongside accelerating progress in our understanding of human physiology at the cellular level. In targeting UCDs, iPSC differentiation into hepatocytes has been the primary focus as the urea cycle is only expressed in totality in this cell type. Optimisation of the hepatocyte differentiation is key in reproducing a defective ureagenesis. Differentiation in additional cell subtypes has enabled to study novel mechanistic approaches and pathophysiology, especially in endothelial cells^67^ and osteoblasts^68^ in ASLD. In the future, iPSC differentiation towards other cell types, particularly involved in the specific pathogenesis of the central nervous system, will be of utmost interest.

iPSCs can be used as a platform for drug discovery, testing different pathways of UCDs for potential therapeutic treatment. For example, induced autophagy has recently been shown to increase ureagenesis, via the cell-penetrating autophagy-inducing Tat-Beclin-1 (TB-1) peptide in OTC and ASL deficiencies when tested in an animal model^105,106^. This approach could be translated to drug screening using iPSCs for other compounds. Drug screening via this approach has been tried in different contexts, including using Sendai virus, and would be amenable to discover novel therapies to restore ureagenesis in UCD or to protect the brain in the context of hyperammonaemia^107,108^.

Advancements in both iPSCs differentiation protocols and CRISPR/Cas9 techniques are continually emerging. For instance, recently researchers have managed to cultivate four organs in a microbioreactor from iPSCs from a single donor. The organ models were positioned in separate compartments of the bioreactor and connected by a microfluidic network^109^. In addition, another study has generated an in vitro whole-organ “Bioreactor grown Artificial Liver Model” (BALM) which enables the long-term 3D culture of iPSC-derived hepatocyte-like cells. This model also allows the transduction of adeno-associated viral and lentiviral vectors, meaning this model could provide a more representative preclinical therapy testing environment^110^. There are also numerous techniques to help identify off-target effects associated with CRISPR/Cas9 editing, such as in vitro and cell-based assays^83,111,112^. These allow off-target effects to be more readily assessed and thus can be taken into account when performing differentiation experiments. Alongside this, the rapid evolution of next-generation sequencing techniques provides means to analyse transcriptomics and chromatin accessibility of both iPSCs and differentiated cell populations. Utilising different multi-omics approaches allows refinement of in-depth analysis and the discovery of novel pathophysiological mechanisms^113^.

## Conclusion

UCD-derived iPSCs are progressively expanding their applications in several areas, from supporting a genetic diagnosis in the genomic era, studying pathophysiology to drug development and validation. Differentiated iPSCs creating patients’ avatars are of particular interest in proof-of-concept studies where they recapitulate key aspects of the disease in a specific disease-enabling genetic and epigenetic background, allowing novel pathophysiological insights. By providing this invaluable opportunity to analyse a genotype of interest within the genetic background of the patient, this allows functional studies in a physiologically relevant model. iPSCs offer the unique opportunity to implement personalised medicine and perform drug screens. The field still faces several challenges, particularly with optimising iPSCs differentiation and successfully translating proof-of-concept studies to effective long-term treatments whilst minimising the risk of adverse effects. While considerable efforts are in place to overcome these challenges, iPSCs present a promising avenue for UCD research and therapy development.
